# Molecular architecture of the uncleaved HIV-1 envelope glycoprotein trimer

**DOI:** 10.1186/1742-4690-10-S1-O1

**Published:** 2013-09-19

**Authors:** Youdong Mao, Luis Castillo-Menendez, Liping Wang, Christopher Gu, Alon Herschhorn, Anik Désormeaux, Andres Finzi, Shi-Hua Xiang, Joseph G Sodroski

**Affiliations:** 1 Department of Cancer Immunology and AIDS, Dana-Farber Cancer Institute, Boston, MA 02215, USA; 2 Department of Microbiology and Immunobiology Harvard Medical School, Boston, MA 02115,USA; 3 Centre de Recherche du Centre Hospitalier de I’Université de Montréal, Department of Microbiology and Immunology, Université de Montréal, Montréal, QC, Canada H3A 2B4; 4 Nebraska Center for Virology, School of Veterinary Medicine and Biomedical Sciences, University of Nebraska-Lincoln, Lincoln, NE 68583, USA; 5 Ragon Institute of Massachusetts General Hospital, Massachusetts Institute of Technology, and Harvard, Cambridge, MA 02139, USA; 6 Department of Immunology and Infectious Diseases, Harvard School of Public Health, Boston, MA 02115, USA

## 

The human immunodeficiency virus (HIV-1) envelope glycoprotein (Env) trimer, a membrane-fusing machine, mediates virus entry into host cells and is the sole virus-specific target for neutralizing antibodies. Binding the receptors, CD4 and CCR5/CXCR4, triggers Env conformational changes from the metastable unliganded state to the fusion-active state. We used cryo-electron microscopy to obtain a 6-Å structure of the membrane-bound, heavily glycosylated HIV-1 Env trimer in its uncleaved and unliganded state. The spatial organization of secondary structure elements reveals that the unliganded conformations of both gp120 and gp41 subunits differ from those induced by receptor binding. The gp120 trimer association domains, which contribute to interprotomer contacts in the unliganded Env trimer, undergo rearrangement upon CD4 binding. In the unliganded Env, intersubunit interactions maintain the gp41 ectodomain helical bundles in a “spring-loaded” conformation distinct from the extended helical coils of the fusion-active state. Quaternary structure regulates the virus-neutralizing potency of antibodies targeting the conserved CD4-binding site on gp120. Recent studies that help validate the 3-D reconstruction of the unliganded HIV-1 Env precursor map will be presented. The Env trimer architecture provides mechanistic insights into the metastability of the unliganded state, receptor-induced conformational changes, and quaternary structure-based strategies for immune evasion.

